# Gastrointestinal tuberculosis following renal transplantation accompanied with septic shock and acute respiratory distress syndrome: a survival case presentation

**DOI:** 10.1186/s12876-017-0695-5

**Published:** 2017-11-28

**Authors:** Andrea Cikova, Diana Vavrincova-Yaghi, Peter Vavrinec, Anna Dobisova, Andrea Gebhardtova, Zora Flassikova, Mark A. Seelen, Robert H. Henning, Aktham Yaghi

**Affiliations:** 1 University Hospital Bratislava, Nemocnica Ruzinov, ICU, KAIM, Clinic of Anesthesiology and Intensive Care Medicine, Faculty of Medicine, Comenius University in Bratislava, Bratislava, Slovakia; 2 Department of Pharmacology and Toxicology, Faculty of Pharmacy, Comenius University in Bratislava, Bratislava, Odbojarov 10, 832 32 Bratislava, Slovakia; 3 Department of Internal Medicine, University of Groningen, University Medical Center Groningen, Groningen, the Netherlands; 4 Department of Clinical Pharmacy and Pharmacology, University of Groningen, University Medical Center Groningen, Groningen, the Netherlands

**Keywords:** Gastrointestinal tuberculosis, Renal transplantation, Multiple organ failure, Acute respiratory distress syndrome, Septic shock

## Abstract

**Background:**

Post-transplant tuberculosis (PTTB) is a serious opportunistic infection in renal graft recipients with a 30-70 fold higher incidence compared to the general population. PTTB occurs most frequently within the first years after transplantation, manifesting as pulmonary or disseminated TB. Gastrointestinal TB (GITB) is a rare and potentially lethal manifestation of PTTB and may show delayed onset in renal transplant recipients due to the use of lower doses of immunosuppressants. Further, non-specificity of symptoms and the common occurrence of GI disorders in transplant recipients may delay diagnosis of GITB.

**Case presentation:**

Here we report a rare survival case of isolated GITB in a renal transplant recipient, occurring seven years after transplantation. The patient’s condition was complicated by severe sepsis with positive blood culture *Staphylococcus haemolyticus*, septic shock, multiple organ failure including acute respiratory distress syndrome (ARDS) and acute renal failure, requiring mechanical ventilation, vasopressor circulatory support and intermittent hemodialysis. Furthermore, nosocomial infections such as invasive aspergillosis and *Pseudomonas aeruginosa* occurred during hospitalization. Antituberculosis therapy (rifampicin, isoniazid, ethambutol and pyrazinamide) was initiated upon *Mycobacterium* confirmation*.* Moreover, treatment with voriconazole due to the *Aspergillus flavus* and meropenem due to the *Pseudomonas aeruginosa* was initiated, the former necessitating discontinuation of rifampicin. After 34 days, the patient was weaned from mechanical ventilation and was discharged to the pulmonary ward, followed by complete recovery.

**Conclusion:**

This case offers a guideline for the clinical management towards survival of GITB in transplant patients, complicated by septic shock and multiple organ failure, including acute renal injury and ARDS.

## Background

Post-transplant tuberculosis (PTTB) is a serious opportunistic infection in renal graft recipients significantly increasing mortality and morbidity [1]. The incidence of PTTB among transplant recipients is 30-70 fold higher compared to the general population, with a significant risk of graft loss and a mortality rate up-to 30% [2]. PTTB is usually the result of reactivation of latent tuberculosis infection, although primary infection or transmission of *Mycobacterium tuberculosis* via the renal graft has been occasionally reported [3]. PTTB occurs most frequently within the first 2 years after transplantation, with a median interval of 8 months [4]. The overall prevalence of PTTB is reported to be 0.3-1.7% in the United States and Western Europe. In developing countries, PTTB prevalence is much higher, ranging between 3.1-15.2% due to the higher prevalence of tuberculosis in those areas [1]. The most common manifestation of PTTB in renal transplant patients is pulmonary TB [5]. One third of all cases of PTTB involve disseminated and extra-pulmonary TB [6]. Amongst the very rare manifestations of PTTB is gastrointestinal TB (GITB) [1]. GITB is potentially lethal [7] and because of its nonspecific symptoms and laboratory findings, diagnosis is often challenging [8]. Moreover, limited data are available regarding the prevalence of GITB among renal transplant recipients [1]. Further, although uncommon, extensive TB of the pulmonary parenchyma, as observed in the course of miliary TB, may cause acute respiratory distress syndrome (ARDS) [9], which is associated with a 60% in-hospital mortality [10]. Mortality increases to 100% when acute kidney injury ensues [11]. So far, a case of post-transplant GITB complicated by ARDS has not been reported.

Here we report a case of isolated GITB in a renal transplant recipient, occurring seven years after transplantation. Moreover, the patient’s condition was complicated by severe sepsis with positive blood culture for *Staphylococcus haemolyticus*, septic shock and multiple organ failure including ARDS and acute renal failure, requiring mechanical ventilation, vasopressor circulatory support and intermittent hemodialysis. Furthermore, nosocomial infections including invasive aspergillosis and *Pseudomonas aeruginosa* occurred during hospitalization.

## Case presentation

A 53-year-old female renal allograft recipient with a history of hypertension presented to the intensive care unit (ICU) with a septic shock and multi-organ failure. In the past, left nephrectomy had been performed due to kidney dysfunction. Seven years before admission, she underwent deceased-donor renal transplantation due to pyelonephritis of the right kidney. The immunosuppressive regimen included methylprednisolone, mycophenolate mofetil (1000 mg-0-1000 mg) and cyclosporine (25 mg-0-50 mg).

Since 7 months, the patient had been re-admitted repeatedly to other hospitals because of recurrent fever, however without demonstration of an infectious agent. One month earlier she had been admitted to the hospital because of dyspnoea and dry cough. Atypical right bronchopneumonia was suspected based on computed tomography (CT) of the lungs and treated empirically with antibiotic therapy including piperacillin/tazobactam, metronidazole, and fluconazole. Since the patient was deteriorating, she was admitted to the pulmonary ICU of our hospital.

### Post-surgery (PO) admission day 1

Because of a history of weight loss and the patient starting to complain of abdominal pain at the pulmonary ICU, and the presence of anemia and intestinal hemorrhage, an abdominal CT was performed. The CT revealed ascites, enlarged uterus with wall thickening, enlarged left ovarium, as well as enlarged mesenteric and retroperitoneal lymph nodes. Further, the low thoracic area showed bilateral pleural effusion and hydropericardium.

Because of the massive intestinal hemorrhage, surgery was needed four days after admission to the pulmonary ICU, during which a right-sided hemicolectomy with side-to-side ileotransverse anastomosis was performed. Additionally, left adnexectomy was performed, because inflammatory tumor lesions were found. As her condition deteriorated post-operatively, the patient was intubated, analgo-sedated to Glasgow Coma Scale 3 (GCS 3) and artificially ventilated with inspired FiO2 0.65-1. Norepinephrine was administered for hypotension. The patient was transferred to our ICU after 24 h of mechanical ventilation in the pulmonary ICU, because of progression of multiple organ failure, including acute renal failure.

**Fig. 1:**
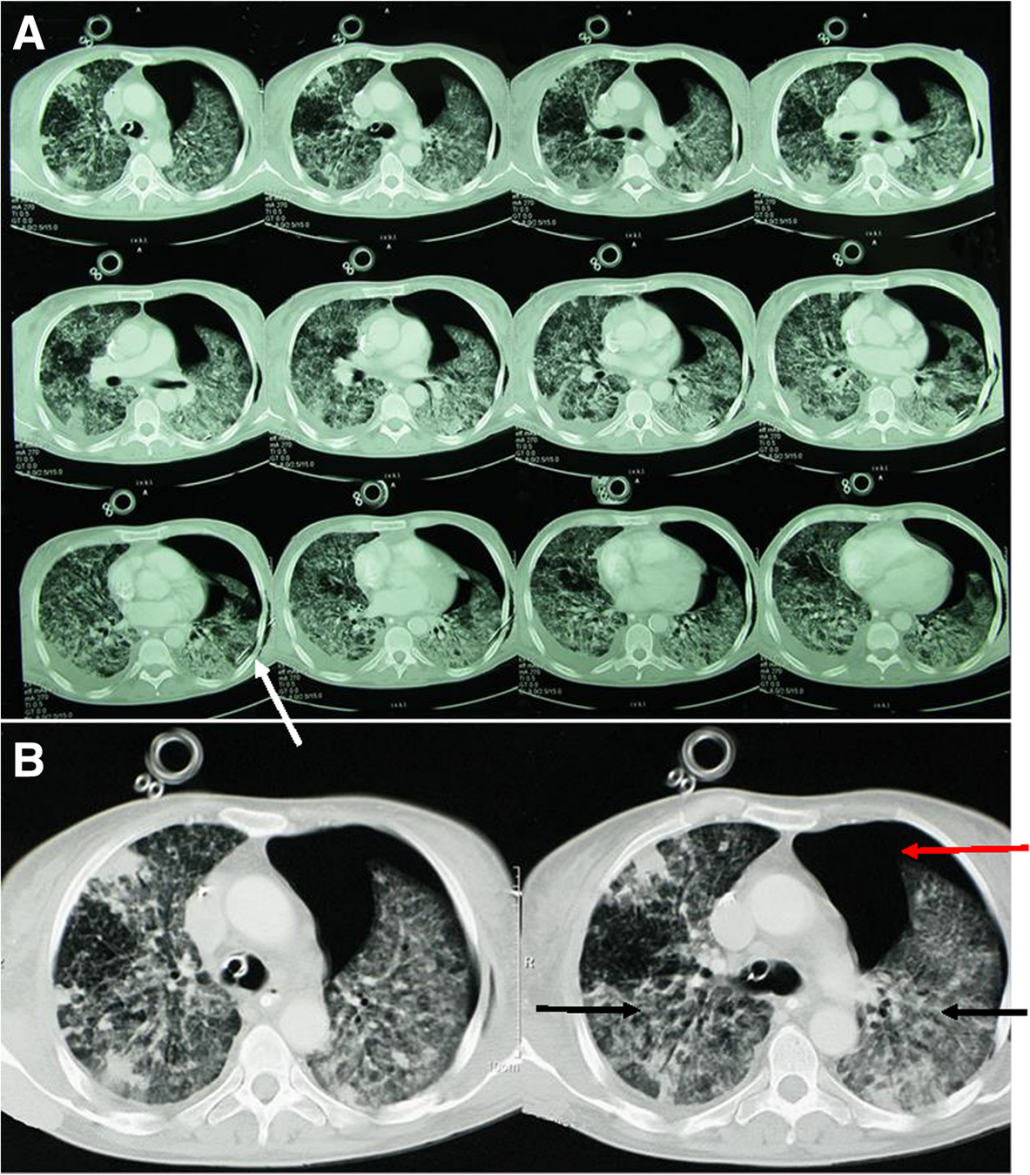
Computer tomography scans at day 1 of admission. CT scan shows acute respiratory distress syndrome (ARDS), diffuse bilateral infiltrates (black arrows), pneumothorax (red arrow) and chest tube (white arrow) (**a**), detailed CT scan (**b**)

On admission at 9:15 a.m., under norepinephrine administration (0.4 μg/kg/min), blood pressure was 110/50 mmHg, HR was 117/min, temperature 37.2 °C, and the patient was anuric. Laboratory analyses revealed combined metabolic and respiratory acidosis (pH 7.136, pCO2 7.8 kPa, BE −11 mmol/l, pO_2_ 12.65 kPa, HCO_3_ 16.1 mmol/l, O_2_ saturation 93.4%, paO2/FiO2 94, arterial lactate 2.13 mmol/l), and hyperkalemia (6.29 mmol/l), hyperchloremia (121.7 mmol/l), elevated blood urea and creatinine (13.11 mmol/l and 179.5 μmol/l, respectively), AST 346.4 U/l and elevated C-reactive protein (196.2 mg/l) and procalcitonin (2.14 μg/l). Blood analyses corroborated massive blood loss (Hb 82 g/l, Ht 0.24, RBC 2.77 × 10^12^/l) and showed WBC 14.2 × 10^9^/l and PLT 147 × 10^9^/l. After the admission, cannulation of left subclavian vein and the left radial artery was performed. Left thoracic drainage was performed due to an iatrogenic left anterior pneumothorax, confirmed by CT, which also showed extensive diffuse bilateral infiltrates (Fig. 1a, b). At 12 a.m., blood culture from a blood sample taken upon admission was found positive for *Staphylococcus haemolyticus.* Moreover, *Staphyloccocus aureus* was cultured from the abdominal cavity. Subsequently, linezolid was added to the therapeutical regimen.

**Fig. 2:**
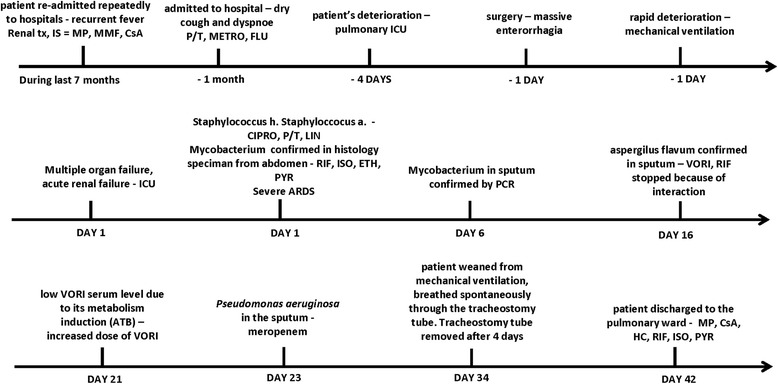
Timeline with essential interventions and medications. tx, transplantation; IS, immunosuppressive regimen; MP, methylprednisolone; MMF, mycophenolate mofetil; CsA, cyclosporine; P/T, piperacillin/tazobactam; METRO, metronidazole; FLU, fluconazole; LIN, linezolid; CIPRO, ciprofloxacin; RIF, rifampicin; ISO, isoniazid; ETH, ethambutol; PYR, pyrazinamide; ARDS, acute respiratory distress syndrome; PCR, polymerase chain reaction; VORI, voriconazole; HC, hydrocortisone; ATB, antituberculosis therapy

Histology tissue specimens obtained from the abdominal cavity revealed granuloma, indicative of tuberculous enteritis, peritonitis, adnexitis and lymphadenitis. Ziehl-Neelsen staining revealed an abundance of gram positive bacterial pathogens type *Mycobacterium*. A final diagnose of systemic mycobacteriosis, with superinfection due to the chronic immunosuppressive therapy was made. Antimycobacterial therapy was initiated with rifampicin, isoniazid, ethambutol and pyrazinamide. Intravenous administration of ciprofloxacin, piperacillin/tazobactam and linezolid was continued. Following the advice of the nephrologist, mycophenolate mofetil was discontinued and immunosuppressive therapy was continued with hydrocortisone. Later that day, the patient was turned to the prone position because of severe ARDS (Fig. 1a, b), for seven hours without improvement in oxygenation (Fig. 2 – timeline with interventions and medications).

### PO day 2 and further

On day 2 of admission to the ICU, oxygenation improved (paO2/FiO2 185), while norepinephrine was continued (up to 0.5 μg/kg/min.). As oliguria persisted, intermittent hemodialysis was indicated (4 h/day). Prior to dialysis, laboratory analyses showed urea 21 mmol/l, creatinine 261.9 μmol/l, potassium 6.22 mmol/l and metabolic acidosis.

Day 3. Intermittent hemodialysis was maintained (4 h/day) and spontaneous diuresis increased to 760 ml/24 h. Norepinephrine infusion rate was gradually lowered.

Day 4. Final intermittent hemodialysis was indicated (4 h/day).

Day 5. Norepinephrine was discontinued.

Day 6. Tracheostomy was performed. *Mycobacterium tuberculosis* in sputum was confirmed by polymerase chain reaction (PCR).

Day 11. Mycophenolate mofetil was re-added to the immunosuppressive regimen.

Day 15. The analyses of sputum revealed filamentous fungi without further specification – the clinical pharmacologist prescribed anidulafungin for yeast superinfection following sensitivity testing (initial dose 200 mg i.v., then 100 mg i.v. every 24 h).

Day 16. Anidulafungin resistant *Aspergillus flavus* was confirmed in sputum, prompting addition of voriconazole to the current treatment (initial dose 300 mg i.v. every 12 h, then 200 mg i.v. every 12 h). The pulmonologist was consulted because of interaction between antimycotic agents and rifampicin. Because of the invasive aspergillosis, rifampicin was stopped.

Day 21. Because of low voriconazole serum level, the dose was increased to 400 mg i.v. every 12 h.

Day 23. Due to the *Pseudomonas aeruginosa* in the sputum, meropenem i.v. was added to the antimicrobial regimen.

Day 34. The patient was weaned from mechanical ventilation and breathed spontaneously through the tracheostomy tube with O_2_. The tracheostomy tube was removed four days later.

Day 42. The patient was discharged to the pulmonary ward, with the following medication: methylprednisolone, cyclosporine, hydrocortisone, rifampicin, isoniazid and pyrazinamide.

## Discussion and conclusions

This case demonstrates both typical and non-typical features associated with GITB in renal transplant patients. First, reactivation of latent TB infection was only observed 7 years after transplantation, which is unusually late, as most cases occur in the first 2 years after transplantation [1]. However, several reports show that the median time of onset of GITB may be longer in renal transplant recipients, possibly due to the lower doses of immunosuppressants in those recipients compared to other transplant recipients [1, 3]. This might have been also the case of our patient, as her immunosuppressive regimen consisted of lower doses of mycophenolate mofetil (1000 mg two times daily) compared to e.g. heart or liver transplant patients (1500 mg two times daily). Moreover, the dose of cyclosporine was decreased several weeks after renal transplantation.

GITB occurs usually due to the reactivation of latent TB infection because of immunosuppressive therapy, or (but less commonly) it is transmitted by the allograft itself; alternatively, it may result from primary infection [3]. Immunosuppressive therapy affecting the function of cell-mediated immunity such as cyclosporine, mycophenolate mofetil and monoclonal and polyclonal antibodies during the first years after transplantation have been associated with TB reactivation [3, 12–14]. Our patient’s immunosuppressive therapy included both cyclosporine and mycophenolate mofetil, however, other risk factors of reactivation (such as pretransplant diabetes, the number of acute rejections of transplanted graft resulting in higher dosing of immunosuppression, opportunistic infections, chronic liver diseases and duration of pre-transplant hemodialysis) were unknown. Interestingly, the receipt of a graft from a deceased donor rather than other sources of donors was recently identified as important risk factor for developing TB in liver or kidney transplant recipients [15], which is in accordance with our case.

Although GITB in our patient occurred most likely due to the latent reactivation, primary infection or transmission via the renal graft cannot be explicitly excluded. However, no recent TB exposure, or significant travel history was reported.

On readmission to the hospital, and subsequent admission to pulmonary ICU, the patient displayed several symptoms commonly found in GITB, including recurrent fever, weight loss, abdominal pain and anemia. The symptoms of GITB in non-transplant patients constitute abdominal pain, weight loss - even anorexia, fever, anemia and bowel habit change [8, 16]. However, in renal transplant recipients, the most common symptoms of decreased inflammatory response due to the immunosuppressive therapy [1, 8] are GI bleeding accompanied by fever and abdominal pain [1]. Furthermore, the symptoms in renal transplant recipients are also often very vague and sometimes plain unusual. Moreover, GI disorders in transplant recipients are relatively common, and are also associated with immunosuppressive therapy or bacterial, viral or parasitic infection [2, 3]. One of the main adverse effects of mycophenolate mofetil is GI irritation [17]. Together, these are causing an important delay in the diagnosis, contributing to the extremely high mortality of renal transplant recipients with GITB. Some authors reported that the late manifestation of GITB in renal transplant recipients is related mainly to this diagnostic delay because of lack of specific symptoms [18]. Therefore, all GI symptoms in renal transplant recipients, although vague, require diagnostic follow up for GITB, albeit confirming the diagnosis may be challenging. CT may often reveal inflammation, ascites (our patient) and lymphadenopathy (our patient). These unspecific features may lead to alternative diagnoses (neoplasms, inflammation or nontuberculosis bacterial or other viral infections or parasites). The microbiological confirmation is often tricky because of difficulties in obtaining the suitable tissue, low sensitivity of microbiological examination and the protracted time needed for culture [19]. Moreover, in immunosuppressed patients skin anergy is frequent; resulting in negative skin tuberculin tests [20] and endoscopic distinction between GITB and other GI diseases (e.g. Crohn’s disease) is difficult. In our patient, tissue specimens obtained from abdominal cavity revealed granulomas with *Mycobacterium*. Six days later, *Mycobacterium tuberculosis* was confirmed by PCR in the sputum. However, CT scan of the lungs revealed no specific lesions, except for atypical right bronchopneumonia. Although we advocate that PCR may be the first line tool in GITB diagnosis, it does not differentiate between an active and latent infection [21]. GITB can coexist with pulmonary involvement; however reports have shown that less than 50% of patients infected with GITB have radiographic evidence of pulmonary disease. A case report of Yilmaz et al. demonstrated renal transplant recipient with GITB, however without X-ray abnormalities [22]. Swallowing of infected sputum in patients with active pulmonary TB belongs to the most common mechanism of GITB origin [23].

In addition, massive intestinal bleeding occurred in line with previous reports showing GI bleeding present in most renal transplant recipients with GITB [1]. As mentioned above, GI bleeding is the most common symptom of posttransplant GITB (in contrast with GITB in a non-transplant population). This could be again due to the decreased inflammatory response because of the immunosuppression; hence the ulcerative disease component is predominant rather than obstructive bowel wall inflammation [1]. Moreover, tissue specimens obtained from the abdominal cavity revealed granuloma, the indicative of tuberculous enteritis, peritonitis, adnexitis and lymphadenitis, which is in line with a previous report [7]. Except for its very late onset, likely due to the low doses of immunosuppressive therapy, and further complications, this case thus represents a typical example of reactivation of latent tuberculosis infection, resulting in GITB with vague abdominal complaints and intestinal bleeding.

Upon observing *Mycobacterium* in tissue obtained from the abdominal cavity, antituberculosis therapy was initiated, consisting of rifampicin, isoniazid, ethambutol and pyrazinamide. However, antituberculosis therapy increases the risk of interactions and toxic hepatitis. Rifampicin is a strong inducer of microsomal enzyme P450 3A4, which leads to increased metabolism of calcineurin-inhibitors, rapamycin and steroids [24]. Rifampicin reduces plasma levels of cyclosporine 2-5 fold and tacrolimus 4-10 fold [1], which increases the risk of rejection [13]. Moreover, common TB medications may lead to interactions with other required drugs, as documented with our case. Therefore, it is strongly recommended to closely monitor and adjust plasma concentrations of drugs, particularly those of the calcineurin-inhibitors, to avoid both toxicity and graft rejection. Therefore mycophenolate mofetil was discontinued for 11 days due to the septic shock (severe infection), while cyclosporine dosage was increased from 25 mg-0-50 mg to 100 mg-0-100 mg. Moreover, our patient’s condition was complicated with aspergillosis and *Pseudomonas aeruginosa* infection. Therefore, voriconazole and meropenem was added to the therapeutic regimen. However, low plasma level of voriconazole was found in the plasma of our patient, likely because of the interaction with rifampicin. Thus, the voriconazole doses also had to be adjusted, while rifampicin was discontinued for one day.

During hospitalization, nosocomial infections including anidulafungin resistant *Aspergillus flavus* and *Pseudomonas aeruginosa* occurred. Immunocompromised patients are more vulnerable for nosocomial infections, especially early after transplantation. On day 1 PO mycophenolate mofetil was discontinued, however on day 11 PO was again re-added to the therapeutic regimen. Subsequently, both *Aspergillus flavus* and *Pseudomonas aeruginosa* was confirmed from the sputum on day 16 and 23 PO. *Aspergillus* infection occurs almost exclusively in immunosuppressed hosts [25], and is associated with increased morbidity and mortality among transplant recipients [26]. *Pseudomonas aeruginosa*, a major nosocomial pathogen, belongs to the life-threatening complication after abdominal organ transplantation. The survival rates are significantly lower for patients with hypotension and on mechanical ventilation, and the predictor of mortality related to *Pseudomonas* bacteremia is the onset of bacteremia in the ICU [27]. Furthermore, the multiple nosocomial infections could be related to previous repeated admission to the hospital or to the prolonged ICU stay (>3 days). In order to reduce risk of nosocomial infection in immunocompromised patients, it is necessary to take preventive measures, such as thorough decontamination and hand hygiene, aseptic procedures, or prophylactic antibiotic therapy. Moreover it is important to reduce the infection risk from use of catheters, cannulas, or tubes. Transplant recipients should receive an empirical antibiotic treatment as well as directed therapy adjusted according to the severity of the infection [28].

Our patient’s condition was furthermore complicated with severe sepsis with *Staphylococcus haemolyticus* blood culture, septic shock, multiple organ failure including ARDS and acute renal failure requiring mechanical ventilation, vasopressor circulatory support and intermittent hemodialysis. Reports of ARDS associated with TB are not scarce; most studies report ARDS to be caused by pulmonary TB, which is associated with in-hospital mortality rates higher than from non-TB causes (e.g. pneumonia) [10, 29]. ARDS may be very rarely caused by miliary TB [10]. To our best knowledge however, there is no case study investigated posttransplant GITB complicated with ARDS.

Although treatable and very rare, GITB is a potentially lethal complication after solid organ transplantation. Onset of GITB is most commonly about 2 years after transplantation, however in renal transplant recipients, with modest immunosuppressive therapy time to manifestation can be longer (our patient). Diagnosis of GITB is challenging, because symptoms are often vague and atypical and renal graft recipients often display GI symptoms. However, early diagnosis is crucial for successful treatment. Therefore, both GI symptoms and fever at any time after transplantation should trigger active diagnostic workup for GITB, especially in the absence of another confirmed infection. Furthermore, (GI)TB treatment in transplant patients poses specific problems because of interactions between antituberculosis drugs and immunosuppressive therapy, especially when other comorbidities such as aspergillosis need to be treated (our patient). Thus, plasma drug levels need to be closely monitored. In case septic shock occurs (our patient), the immunosuppressive therapy may be discontinued. Moreover, GITB could be complicated with multiple organ failure including ARDS and acute renal failure, which may necessitate mechanical ventilation, vasopressor circulatory support and hemodialysis. Nevertheless, the case currently presented demonstrates that by implementing the above recommendations, survival from GITB complicated by septic shock and multiple organ failure including acute renal injury and ARDS may be accomplished.

## Abbreviations

ARDS: Acute respiratory distress syndrome
AST: Aspartate aminotransferase
ATB: Antituberculosis therapy
CIPRO: Ciprofloxacin
CsA: Cyclosporine
CT: Computed tomography
ETH: Ethambutol
FLU: Fluconazole
GCS 3: Glasgow Coma Scale
GITB: Gastrointestinal tuberculosis
Hb: Hemoglobin
HC: Hydrocortisone
HR: Heart rate
Ht: Hematocrit
ICU: Intensive care unit
IS: Immunosuppressive regimen
ISO: Isoniazid
LIN: Linezolid
METRO: Metronidazole
MMF: Mycophenolate mofetil
MP: Methylprednisolone
P/T: Piperacillin/tazobactam
PCR: Polymerase chain reaction
PLT: Platelets
PO: Post-surgery
PTTB: Post-transplant tuberculosis
PYR: Pyrazinamide
RBC: Red blood cells
RIF: Rifampicin
TB: Tuberculosis
TX: Transplantation
VORI: Voriconazole
WBC: White blood cells
